# Evaluation of Financial Support Workshops for Patients Under State Pension Age With Degenerative Cervical Myelopathy: Survey Study

**DOI:** 10.2196/59032

**Published:** 2025-02-24

**Authors:** Tanzil Rujeedawa, Zahabiya Karimi, Helen Wood, Irina Sangeorzan, Roy Smith, Iwan Sadler, Esther Martin-Moore, Adrian Gardner, Andreas K Demetriades, Rohitashwa Sinha, Gordan Grahovac, Antony Bateman, Naomi Deakin, Benjamin Davies

**Affiliations:** 1 Division of Neurosurgery, Department of Clinical Neurosciences, University of Cambridge, The Old Schools, Trinity Ln, Cambridge, CB2 1TN, United Kingdom, 01223 337733; 2 Myelopathy.org, Cambridge, United Kingdom; 3 Aston University, Birmingham, United Kingdom; 4 Edinburgh Spinal Surgery Outcome Studies Group, Department of Neurosurgery, Royal Infirmary Edinburgh, Edinburgh, United Kingdom; 5 Department of Neurosurgery, Leeds General Infirmary, Leeds, United Kingdom; 6 King's College Hospital, London, United Kingdom; 7 Royal Derby Spinal Centre, Derby, United Kingdom

**Keywords:** myelopathy, degenerative, spine, spinal, benefits, aid, financial, money, income, poverty, disability, disabled, finance, workshop, education, service, access, accessibility, navigate, confidence, government

## Abstract

**Background:**

Degenerative cervical myelopathy (DCM), a form of slow-motion and progressive spinal cord injury caused by spinal cord compression secondary to degenerative pathology, leads to high levels of disability and dependence, and may reduce quality of life. Myelopathy.org is the first global scientific and clinical charity for DCM, providing an accessible platform freely disseminating information relevant to the DCM diagnosis and its treatment. Significant transient and long-term change to earnings do occur and can thrust individuals into poverty. People with DCM face many challenges accessing state financial assistance. This can have a cumulative negative financial effect due to the association between DCM and low socioeconomic index. Financial support available to patients under pension age include Universal Credit (UC), a payment that helps with living costs, and Personal Independence Payment (PIP), which helps with extra living costs if someone has both a long-term health condition or disability and difficulty doing certain everyday tasks.

**Objective:**

This study aimed to assess if delivering workshops centered around access to financial support could assist people with DCM living in the United Kingdom.

**Methods:**

A series of 2 internet-based workshops was targeted at accessing financial support for English patients under the state pension age, with an anonymized survey delivered to participants after each session. The first session was on UC and the second on PIP. The survey consisted of a mixture of Likert scales, free text and yes or no answers. Survey responses were analyzed using descriptive statistics and free text answers underwent inductive thematic analysis.

**Results:**

The average rating on the use of UC was 9.00/10. Presession self-rated confidence levels were 5.11/10 rising to 8.00/10. The mean score of wanting further similar sessions was 8.67/10 with 56% (5/9) of participants wanting one-to-one sessions. For PIP, the average session use rating was 10/10. Presession self-rated confidence levels were 4.43/10 rising to 9.57/10. The mean score of wanting further similar sessions was 8.71/10, with 43% (3/7) of participants wanting one-to-one sessions . Following inductive thematic analysis, themes regarding the usefulness of such sessions and the challenges to accessing financial support emerged. One participant gave negative feedback, which included the length of the session and perceived problems around confidentiality and data protection.

**Conclusions:**

The pilot series was largely perceived as a success, with participants finding them useful and increasing their self-rated confidence in navigating the UK financial support system. Given the small sample size, it is hard to predict the success of future sessions. Finally, given that the hurdles in accessing financial support extend beyond DCM, such workshops may be relevant to other organizations.

## Introduction

Degenerative cervical myelopathy (DCM) is a form of slow-motion and progressive spinal cord injury caused by spinal cord compression secondary to degenerative pathology. This includes disease processes such as cervical spondylosis, ossification of the posterior longitudinal ligament, ossification of the ligamentum flavum, and degenerative disc disease [1,2]. Globally, it is estimated to affect 2% of adults, although less than 10% are formally diagnosed at this time [3,4]. DCM can cause sensorimotor dysfunction in the upper or lower limbs, gait disturbance, bladder or bowel dysfunction, and pain [2,5-9]. Surgery is the mainstay of treatment for DCM and aims to decompress the spinal cord [2,10-12]. Whilst this typically stops further deterioration and may afford meaningful symptomatic improvement, recovery is generally incomplete. People with DCM experience high levels of disability and dependence, and are among those with the lowest quality of life scores for any chronic disease [13]. In fact, almost half of diagnosed individuals may be dependent on the support of others for activities of daily living (41.9% (326/778) in a recent study [14]).

This landscape, in the study mentioned above, results in great socioeconomic impact, with over a third of participants unable to return to work (35.7%; 278/778 of people with DCM [14]) due to DCM. Furthermore, 25.8% (201/778) were employed full time and 11.3%(88/778) were employed part-time [14]. In addition, 35.7% (278/778) were unable to work due to their disability [14]. Surgery can be helpful, with Lønne et al [15] showing that by 12 months after surgery, 65% (589/906) had returned to work, increasing to 75% (680/906) by 36 months. Clearly, therefore, DCM impairs patients’ ability to contribute economically before retirement and can lead people into poverty [16]. Davies et al [17] found that, based on the average age of cases at presentation in the United Kingdom and an average age of retirement of 65 years, there is a potentially 15.1 years of affected productivity in people with DCM [17]. This equates to an estimated lifetime inflated loss of income of up to £347,112 (US $435,150) using UK Office for National Statistics figures or £474,719 (US $595,121) using Office for Economic Co-Operation figures [17]. In fact, there is an estimated annual loss of productivity of £362.6m (US $453.3m), in addition to disability financial support totaling £280.2m (US $350.3m) and an overall cost to society for this cohort of £681.6m (US $ 853.9m) in the United Kingdom [17]. This has a cumulative negative financial effect due to the association of DCM with a low socioeconomic index, a delayed diagnosis, and poor outcomes [18].

Whilst this provides an estimate of the current “demand” and potential future “societal cost” of DCM, the practicalities of ensuring that people with DCM can access support is seldom straightforward, since social support systems differ by both jurisdiction and patient circumstance. A study from Norway, where financial support is available to all citizens suffering ill-health, found that 20% of patients received medical income compensation financial support 1 year before surgery, rising to 100% at the time of surgery. However, for most countries, this “gold standard” access is complex. First, the term “DCM” is relatively new, having been introduced in 2015 [1]. Furthermore, being focused on undergoing treatment and lacking a true understanding of the long-term implications of the disease, people with DCM rarely recognize (or accept) the need for financial support until needs are heightened [19-21]. Professionals may be aware of these challenges, as patients often turn to their medical team for support, guidance, and written opinions. However, many health care professionals are not trained to advise patients on available options and are likely unequipped to best navigate the claim and assessment process.

Myelopathy.org is the first global scientific and clinical charity for DCM. The charity provides an accessible internet-based platform freely disseminating information relevant to the DCM diagnosis and its treatment. As an organization, it hosts peer-to-peer support forums, and fields inquiries from those affected by DCM. Across this media, contacts requesting information or assistance for accessing financial support is a frequent topic.

Several active organizations exist that offer advice and guidance to people applying for financial support. These include Hastings Advice and Representation Centre (HARC) [22], Citizens Advice [23], and Welfare Benefits Unit [24]. In fact, certain charities such as the British Heart Foundation have dedicated web pages specific to applying for financial support [25]. Some, such as Age Scotland, offer workshops to their beneficiaries about accessing benefits [26]. However, there is no literature assessing the utility of these interventions. We hypothesized that delivering workshops centered around access to financial support could assist people with DCM.

## Methods

### Overview

The authors, in collaboration with Myelopathy.org, piloted a series of 2 workshops in May 2023 targeted at accessing financial support for English patients under the state pension age.

### Participants

People with DCM were recruited to the sessions virtually, using an advertisement within the Facebook (Meta) support group run by Myelopathy.org, which had 2065 members at the time of the study. Interested participants contacted the charity directly by email to book sessions. This was the only form of recruitment. Inclusion criteria was any person with DCM under state pension age from England. Where a session was oversubscribed, a random number generator was used to select 10 participants.

### Procedures

The sessions were delivered in a paid partnership with HARC, a charitable organization, which provides welfare financial support advice and information to the people of East Sussex and the South Coast of England [22]. It aims to address inequalities, relieve poverty, and improve the quality of life for vulnerable and disadvantaged individuals and families [22]. Funding was from the National Lottery community fund, a UK specific fund [27].

The overall aim was to equip participants with the skills to navigate the financial support system independently. The workshop program consisted of 2 sessions delivered as a set. All sessions were held virtually on Zoom (Zoom Video Communications) and each lasted up to 2 hours. The workshop format was chosen in partnership with HARC. HARC provided expertise from generic sessions delivered previously, with the chosen workshop format encouraging interactivity and the ability to ask questions, as well as being cost-effective.

The sessions were targeted to patients currently under state pension age, allowing content to be tailored to age demographics. This is dependent on when someone was born [28]. The first session was on Universal Credit (UC), a payment that helps with living costs [28] and the second on Personal Independence Payment (PIP), which helps with extra living costs if someone has both a long-term health condition or disability and difficulty doing certain everyday tasks or getting [29]. The sessions will be termed UC and PIP, respectively. Each session consisted of a comprehensive overview of either UC or PIP and their application process with a question time at the end.

### Measures

Participants were evaluated using an anonymized survey (Google Forms), sent by email to the participants by the charity after attending each workshop.

### Analysis

Survey responses were analyzed using descriptive statistics (mean, median, mode, and range) using R (version 4.2.1; R Foundation for Statistical Computing). Free text answers underwent inductive thematic analysis to identify key themes of feedback by TR and BD separately. Themes were identified manually with TR an BD having a discussion afterwards to ensure validity. Given the small quantity of data, no codebook software was used.

### Ethical Considerations

This study was conducted with ethical approval from the Myelopathy.org Board (M2023001). At the start of the workshop, participants were provided with an overview of the workshop and that feedback would be asked at the end that may be used for publication. By continuing into the workshop, participants were providing informed consent to participate. All data collected were anonymous. No incentives were offered for completion of the surveys.

## Results

A total of 24 people with DCM registered for the workshops. There were 9 attendees in UC and 7 in PIP, with 2 individuals dropping out from the first session to the second. Demographic information was not recorded.

### Quantitative Feedback

**Figure 1.**
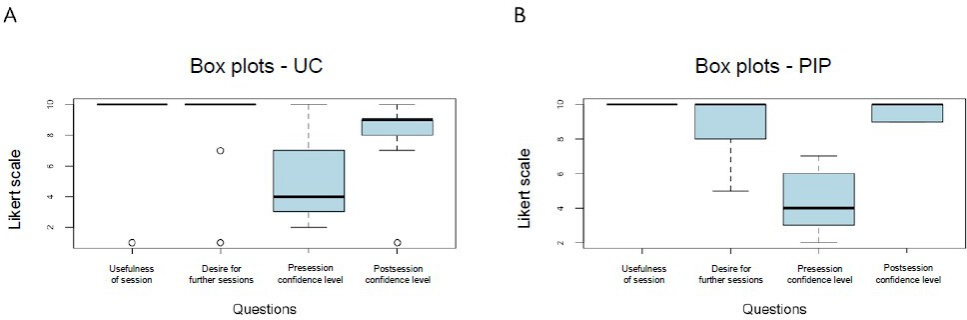
A. Box plots showing the response in the survey administered after the UC session. B. Box plots showing the response in the survey administered after the PIP session. UC: universal credit; PIP: personal independence payment.

Participants were asked to rate the sessions across four questions, using a Likert scale (1 to 10) for each. Figure 1 summarizes the responses to the survey administered after each session, while Tables S1 and S2 (Multimedia Appendix 1) provide responses at the level of each participant.

The average rating on the usefulness of UC was 9.00/10 (SD 2.82). Presession self-rated confidence levels were 5.11/10 (SD 2.73) rising to 8.00/10 (SD 2.62) after the session. The mean score of wanting further similar sessions was 8.67/10 (SD 2.87), with 56% (5/9) of participants wanting one-to-one sessions.

For PIP, the average session usefulness rating was 10/10 (SD 0), with the desire for further sessions. Presession confidence levels were 4.43/10 (SD 1.76) rising to 9.57/10 (SD 0.49). The mean score of wanting further similar sessions was 8.71/10 (SD 1.75) with 43% (3/7) of participants wanting one-to-one sessions.

### Qualitative Feedback

Several themes emerged during the thematic analysis, notably with regards to the usefulness of the sessions and the challenges to accessing financial support.

An important theme that emerged was the usefulness of the sessions. Participants noted that the sessions were “interactive,” “amazing,” “informative,” and that they had “enjoyed this very much.” Both sessions were generally viewed as very helpful, with several participants noting the information given being particularly useful with a participant noting that the sessions contained “specific information that was tailored to participants’ needs, rather than just a bullet point description of financial support available.” The information on what the questions on the forms actually meant and how to answer them was noted as especially helpful. In fact, most participants noted that they could not suggest any improvements for the sessions. The desire for further sessions was also noted, with several participants keen for more as demonstrated by one of the participants saying that they “would like more of these types of sessions as they are so so beneficial and also make you feel like you are less alone in the journey,” and another saying “just more sessions would be amazing*.*” One participant even suggested group discussions that include Myelopathy.org members who had made successful claims.

The challenges to accessing financial support were also highlighted. The confusing system was the main challenge noted. The lack of knowledge about eligibility, lack of understanding and knowledge from professionals and fear of judgment were also mentioned. One participant “felt alone and like I was being judged” and another “never applied before because I never felt like I was entitled to any, but I feel more confident now.” In addition, participants found it hard to understand how detailed answers to the forms needed to be. Finally, physical disability making it hard to fill in forms was another issue.

Certain negative themes also came across from only 1 of the participants. They did not find the session helpful due to what was perceived as “unnecessary personal introductions, and waffle,” and some technical issues that prevented the screen from being shared properly at the start. In addition, they felt that details of the session were not properly explained in advance. They also perceived that the facilitator had strayed “into areas of confidentiality and data protection.” This was due to an incident whereby another one of the participants had mentioned to the facilitator that they were going to the toilet and the facilitator accidentally mentioned it to the whole group. The participant involved had no issues with this.

## Discussion

With the support of a charitable partner, Myelopathy.org delivered a series of workshops that were effective in increasing awareness and confidence in obtaining financial support in the United Kingdom. No other similar study was found through a search on PubMed for comparison, even in a non-myelopathy context, thereby illustrating the novel nature of this initiative. Workshops were considered highly useful (9-10/10) and increased confidence by >40%.

Following inductive thematic analysis, themes regarding the usefulness of the sessions and the challenges to accessing financial support emerged. The interactivity of the session was viewed as an important positive. The usefulness of the sessions was also noted as well as the desire for further sessions. The challenges of applying for financial support were also explored, notably the confusing system, physical disability, the lack of knowledge about eligibility, lack of understanding and knowledge from professionals, and fear of judgment. One participant had negative feedback, which included the length of the session and perceived problems around confidentiality and data protection. The feedback was taken seriously and given to the facilitator. An apology was presented to the participant by ZK, the director of Myelopathy.org, and the misunderstanding was resolved. This participant did not attend PIP and could represent a subgroup of the people with DCM population for whom another mode of engagement would be more suitable such as anonymized access of online web pages or documents.

While the intervention was considered successful, it is important to consider areas for improvement, particularly to ensure the intervention can be sustained and exported to other regions or countries. Of note, participants were split about wanting further one-to-one sessions despite thinking further sessions would be helpful. This could be potentially explained by the participants preferring to be in a group setting rather than alone.

Experience from other settings is limited and is an avenue that should be explored. It should also be acknowledged that individuals who sign up for a group workshop may be more likely to like such settings. This initial evaluation also does not collect objective data relevant to actual or successful financial support applications. Other limitations include the fact that the sessions were focused solely on financial support available in England and particular economic situations. How these findings relate to outside England and situations such as self-employed status is therefore unclear. Furthermore, delivery of sessions virtually, available to only a limited number of participants, may provide only a narrow snapshot of people with DCM. This situation is compounded by the fact that Facebook-only recruitment may skew participant demographics. Another limitation is that no demographic data were collected that could have helped in contextualizing findings and identifying possible trends across specific demographic groups.

Despite the limitations outlined above, the participants largely deemed this initial pilot to be a welcome success. Given this success, Myelopathy.org aims to provide further similar sessions both in England and other countries. In addition, this can serve as a template for small charities focusing on relatively unknown conditions that cause a financial burden. The socioeconomic impact of DCM is a global research priority [30]. The chronic disability experienced by most, even if transiently during the drawn-out diagnostic process and aftermath from surgery, can dramatically alter household incomes and thrust people into poverty [16]. There is a cumulative negative effect due to the association of DCM with low socioeconomic conditions.

### Conclusion

Despite the presented limitations and a small amount of negative feedback from one individual, the sessions were largely perceived favorably, with participants finding them useful and increasing their self-rated confidence in navigating the UK financial support system. The small sample size limits any further extrapolation; it is indeed hard to predict how future sessions will be received. Nonetheless, Myelopathy.org intends to co-host future sessions with the lessons learned from this pilot. Finally, difficulties in accessing financial support extend beyond DCM, and hence, the lessons learned in this evaluation may be helpful to other patient groups and support organizations.

## Supplementary material

10.2196/59032 Multimedia Appendix 1 Supplementary tables.

## Abbreviations

DCM: degenerative cervical myelopathy
HARC: Hastings Advice and Representation Centre
PIP: personal independence payment
UC: universal credit
