# Supplementary material for: Evaluation of Financial Support Workshops for Patients Under State Pension Age With Degenerative Cervical Myelopathy: Survey Study
Source: JMIR Form Res. 2025 Feb 24;9:e59032. doi: 10.2196/59032 (PMC11875102; doi:10.2196/59032)
Supplement: Multimedia Appendix 1 [file formative-v9-e59032-s001.docx]

**Table S1**

Table S1: Post-UC participant-level survey responses and descriptive analysis

| **Question** | **Participant** | | | | | | | | | **Mean** | **Median** | **Mode** | **Range** |
| --- | --- | --- | --- | --- | --- | --- | --- | --- | --- | --- | --- | --- | --- |
|  | **1** | **2** | **3** | **4** | **5** | **6** | **7** | **8** | **9** |  |  |  |  |
| Please rate how useful the session was. | 10 | 10 | 10 | 10 | 10 | 10 | 10 | 10 | 1 | 9.00 | 10 | 10 | 9 |
| How useful would more in-depth sessions walking you through the whole process be? | 10 | 10 | 10 | 10 | 10 | 7 | 10 | 10 | 1 | 8.67 | 10 | 10 | 9 |
| Please rate on a scale of 1 to 10 how confident you felt navigating the financial support system before the session. | 9 | 5 | 10 | 2 | 3 | 3 | 3 | 4 | 7 | 5.11 | 4 | 3 | 8 |
| Please rate on a scale of 1 to 10 how confident you now feel navigating the financial support system after the session. | 9 | 10 | 10 | 9 | 8 | 7 | 9 | 9 | 1 | 8.00 | 9 | 9 | 9 |

**Table S2**

Table S2: Post-PIP participant-level survey responses and descriptive analysis

| **Question** | **Participant** | | | | | | | **Mean** | **Median** | **Mode** | **Range** |
| --- | --- | --- | --- | --- | --- | --- | --- | --- | --- | --- | --- |
|  | **1** | **2** | **3** | **4** | **5** | **6** | **7** |  |  |  |  |
| Please rate how useful the session was. | 10 | 10 | 10 | 10 | 10 | 10 | 10 | 10 | 10 | 10 | 0 |
| How useful would more in-depth sessions walking you through the whole process be? | 8 | 10 | 10 | 5 | 10 | 10 | 8 | 8.71 | 10 | 10 | 5 |
| Please rate on a scale of 1 to 10 how confident you felt navigating the financial support system before the session. | 2 | 3 | 3 | 4 | 6 | 6 | 7 | 4.43 | 4 | 3, 6 | 5 |
| Please rate on a scale of 1 to 10 how confident you now feel navigating the financial support system after the session. | 10 | 9 | 9 | 10 | 9 | 10 | 10 | 9.57 | 10 | 10 | 1 |
